# Illness acceptance, pain perception and expectations for physicians of the elderly in Poland

**DOI:** 10.1186/s12877-017-0441-4

**Published:** 2017-02-08

**Authors:** Mateusz Cybulski, Lukasz Cybulski, Elzbieta Krajewska-Kulak, Urszula Cwalina

**Affiliations:** 1 Department of Integrated Medical Care, Faculty of Health Sciences, Medical University of Bialystok, 7a M. Sklodowskiej-Curie str., 15-096 Bialystok, Poland; 2 National security student, Faculty of Social Sciences, University of Warmia and Mazury in Olsztyn, Olsztyn, Poland; 3 Department of Statistics and Medical Informatics, Faculty of Health Sciences, Medical University of Bialystok, Bialystok, Poland

**Keywords:** Illness acceptance, Pain control, A list of a patient’s expectations, The elderly, Old age, Psychology of ageing

## Abstract

**Background:**

Ageing of society is a significant challenge to public health, both socially and health wise. Adaptation to illness and its acceptance play an important role in control and patients’ self-control in many diseases of old age. The right attitude of doctors to patients, especially, geriatric patients determines, among others, a patient’s quality of life and acceptance of illness. Recently, there has been observed the rapid development of research on interactions between pain as a physiological process and its perception by an individual.

The aim of the study was to evaluate the acceptance of illness, perception of pain and expectations of geriatric patients for physicians among the inhabitants of Bialystok (Poland) over the age of 60.

**Methods:**

The study included 300 people, inhabitants of Bialystok and the surrounding area – aged over 60: 100 elderly residents of a nursing home, 100 students of the University of the Third Age in Bialystok and 100 students of the University of Healthy Senior. The study used three standardised psychometric scales: Patient Request Form (PRF), Acceptance of Illness Scale (AIS) and The Beliefs about Pain Control Questionnaire (BPCQ).

**Results:**

The median of the overall score of AIS was 26 points, which is considered average in terms of acceptance of illness. The median value of the influence of internal factors on the control of pain in case of BPCQ scale was generally16 of 30 points, the influence of physicians – 15 of 24 points, while random events – 14 of 24 points. The overall result for PRF scale proved that the respondents were the least expected to look for emotional support (5 of 12 points). It was established that the group affiliation significantly affected the result of AIS (*p* < 0.001). There was also noted a negative relation between AIS and the search for emotional support (PRF) depending on the group. The higher the AIS value, the lower the score in case of search for emotional support (PRF).

**Conclusions:**

Neither gender nor age played a significant role in acceptance of illness, control of pain or expectations for physicians. The key variable determining the occurrence of dependencies between the studied features was being a part of a group. The elderly residents of the nursing home were negatively distinguished from the other two studied groups. The respondents, in regard to other groups described in the literature, were characterised by relatively high values in illness acceptance, pain control and expectations for physicians.

## Background

Population ageing is a significant challenge to public health, both socially and health wise. It is estimated that by 2050, in the countries of medium and low income, 80% of the whole population will be over 60. In the analyses of the World Health Organization (WHO), Japan is indicated as an example of the country where as many as 30% of the population is presently over 60 [1]. By 2050 year, similar rates will be observed in Chile, China, Iran and Thailand [1], while people over the age of 65 will constitute more than 25% of the whole European population [2]. By 2020, more than million Poles will be 90 years old, and by 2035 more than 25% of Poles will be 65 years old and over. In 2060, Poles will be one of the oldest populations in Europe [3].

Adaptation to illness and its acceptance play a significant role in control and a patient’s self-control in diseases present in old age. Acceptance of the disease greatly affects the self-esteem and adaptation to limitations determining a subjective life quality of the elderly [4]. In the reference literature [5, 6] it has been proved that the higher the level of illness acceptance, the weaker the negative reactions and emotions associated with the disease and its treatment. The results have also shown that the residents of nursing homes were characterized by a lower level of illness acceptance than the seniors living with their families [7].

The right attitude of physicians to patients, especially, geriatric patients determines, among others, illness acceptance and patients’ quality of life. Therefore, apart from the direct provision of health care, the contact between patients and medical staff should also include, information about the health status, course of the disease, ways of treatment, possible side effects and their types, and most importantly, it should lead to establishing and maintaining emotional contacts through empathy and kindness in direct relations between patients and medical personnel [8]. The quality of such contact plays an important role in shaping the appropriate attitude towards illness and its treatment and influences mental wellbeing and mobilization to fighting the disease [9]. An active contact with a physician gives a patient a greater sense of security and increases his trust in the physician, which contributes to motivation for treatment [10]. Therefore, the study of a patient’s expectations for physicians is an important and underestimated niche for examination of seniors’ life quality.

Recently, the rapid development of research on interactions between pain as a physiological process and its perception by an individual has been observed [11–13]. Pain is characterised by two aspects: the first – a sensory function, responsible directly for the sensation of pain. It enables to locate the pain. The second- an emotional aspect, which is characterised by psychological reaction to the pain stimulus. The emotional component of pain is most often a subjective sensation, therefore, the feeling of pain is different in individual people, especially, in the elderly [14–16]. A sense of control over the pain and self-efficacy are significant determinants of pain sensation [17]. The studies of pain sensation are most frequently conducted among cancer patients; only a few studies referring to the subjective sensation of pain and ways of dealing with it have been carried out among the elderly, who usually suffer from multiple morbidities.

The aim of the study was to assess illness acceptance, pain perception and a geriatric patient’s list of expectations for physicians among Bialystok inhabitants aged over 60.

## Methods

### Participants

The study was carried out in three groups:

- I group – students of the University of Healthy Senior (UHS) (100 people), held at the Faculty of Health Sciences of the Medical University of Bialystok, and aimed at promoting healthy lifestyle and attitude, broadening the knowledge of health sciences, as well as social activation and prevention of loneliness among the elderly. The course was based on three main pillars, i.e. lectures, practical classes and optional classes. The classes included only health-related topics;
- II group – students of the University of the Third Age (UTA) in Bialystok (100 people), the aim was the education and stimulation mainly of the retired members of the community. Typical courses included art, classical studies, conversation, computers, crafts, debate, drama, history, languages, literature, music, sciences, social sciences, and philosophy. There were also many less educationally focused activities, including games, health, fitness and leisure, theatre/concert clubs, travel clubs and dance in all forms;
- III group – residents of the Nursing Home (PNH) at 9 Swierkowa Str. in Bialystok (100 people).

None of the students of the Universities was a resident of the nursing home.

In total, the study included 300 people aged over 60 – inhabitants of Bialystok and the surrounding areas.

An additional criterion for inclusion in the study, apart from the age and place of residence, was lack of confirmed dementia in potential respondents. Each of the participants had to give written consent to the participation in the study and could withdraw from it at any stage.

Similarly, the criteria excluding from the study were: the age below 60, the place of residence outside of Bialystok and its surrounding areas, as well as the occurrence of dementia.

The selection of respondents was intended. For the purpose of the study the authors collected 100 complete surveys in each subgroup. Among the students of the University of the Third Age and the University of Healthy Senior more copies of the research tools were distributed, but not all distributed questionnaires returned to the authors of the study. Among the students of the University of Healthy Senior there were distributed approx. 150 copies of questionnaires, and among the participants of the University of Third Age - 200 copies.

### Measurements and procedure

The study used three standardised psychometric scales:

- Patient Request Form (PRF) by P. Salmon and J. Quine, adapted by Z. Juczynski;
- Acceptance of Illness Scale (AIS) by B. J. Felton, T. A. Revenson and G. A. Hinrichsen, adapted by Z. Juczynski;
- The Beliefs about Pain Control Questionnaire (BPCQ) by S. Skevington, adapted by Z. Juczynski.

Statements included in AIS express defined difficulties and limitations caused by the health status. A degree of illness acceptance is expressed by lack of negative reactions and emotions connected with the disease. Acceptance of Illness Scale can be used to measure acceptance of every illness. The scale contains eight questions describing consequences of bad health state, regarding limitations caused by the disease, lack of self-sufficiency, a sense of dependence on others and lowered self-esteem. In every statement, a respondent defines his/her present status using a five-degree Likert’s scale (from 1 – ‘I strongly agree’, to 5 – ‘I strongly disagree’). Strong agreement (1 point) expresses negative adaptation to illness and considerable mental discomfort, whereas strong disagreement (5 points) means illness acceptance. The overall result ranges from 8 to 40 points. The higher the score, the greater the acceptance of health status and less negative emotions associated with the disease The overall result of less than 20 points is regarded as low, while the result above 30 points means a high level of acceptance of one’s own health status, and the result of 20 to 30 points is regarded as average. Interpretation of the results obtained is based on their comparison with mean indicators in different groups of patients. The Cronbach alpha coefficient for this method equals 0.82 [18].

PRF is a tool of self-description and consists of 18 statements relating to various reasons for visiting a general practitioner. The respondent indicates to what extension ‘the statement expresses the reasons for visiting a physician. The statements are part of three factors, such as the explanation of the disease, seeking emotional support and obtaining information about examinations and treatment. The results of PRF relating to the explanation of the disease are associated with expectation of help from general practitioners, while the results regarding obtaining the information on examinations and treatment – expectation of help from physicians. In turn, the expectation of emotional support is connected with the emphasis on the importance of counselling, as well as psychiatric or psychological help. For each statement, one should choose one of the three answers relating to reasons for visiting one’s physician: yes - if the respondent agrees with the statement; I am not sure – if the respondent is not sure; no – if the respondent does not agree with the statement. All questions shall be answered. The average time for answering the questions does not exceed 10 min. The answer of agreement to the statement gives 2 points, uncertainty – 1 point, while the negative answer – 0 points. The results for each factor are assessed separately. The theoretical range of results in each of the three scales is from 0 to 12 points. The higher the score, the greater the expectations of a specified type of aid [18]. For the purposes of this study the questionnaire has been modified, by adding the statement about the last visit to the doctor, not today’s visit as it is in the original version.

BPCQ refers to the scales measuring locus of control, also health control. It is used to assess the strength of individual beliefs about pain control by means of:

1. internal factors;
2. influence of physicians (others’ strength);
3. random events.

The Cronbach alpha coefficient equals 0.75 for the whole scale, and for the subscales: influence of physicians (0.86), internal locus of pain control (0.82), influence of incidents (0.58). BPCQ is used to examine adult patients, complaining about pain. It can also be used to measure beliefs about pain control in people who currently do not suffer from pain. The questionnaire can be used in the diagnosis and therapy of outpatients and inpatients. The questionnaire is based on the assessment of the given opinions in a 6-level Likert’s:

1. No, I completely disagree;
2. I disagree;
3. I rather disagree;
4. I rather agree;
5. I agree;
6. Yes, I completely agree.

The results cannot be presented as a single indicator. The sum of results is calculated separately for each aspect of pain control locus. The range of possible points is 5 to 30 points – in regard to internal control and 4 to 24 points – for the other two aspects. A higher result reflects a stronger belief that pain can be controlled with the influence of one factor [18].

After the members of the research team gave a detailed explanation of the study procedure, the respondents from group I to II completed the questionnaires independently. Additional explanations and instructions were also included in every questionnaire of the scale. In group III (the residents of nursing home), the respondents were directly interviewed by psychologists and occupational therapists employed by the institution. The survey among the residents of nursing home had a form of interviews, as they usually were not highly educated, due to which they may not have been able to read and fill out questionnaires independently. In addition, the vast majority of the residents were chronically ill.

### Procedure and ethical considerations

The study was conducted from February to June 2016. The research conformed with the Good Clinical Practice guidelines and the procedures were in accordance with the Helsinki Declaration of 1975, as revised in 2000 (concerning the ethical principles for medical research involving human subjects and prohibiting the provision of patient’s name, initials or hospital evidence number) and with the ethical standards of the institutional committee on human experimentation (statute from the Bioethics Committee of the Medical University in Bialystok no. R-I-002/365/2015). The members of the research team gave written and verbal information about the study to potential participants. They receivded the information about the project and gave written consent to participate.

### Statistical analysis

The data were processed using Microsoft Excel 2013 and statistically analysed by means of Statistica Data Miner C QC PL. Pearson’s Chi-square (χ^2^) test was used to analyse the dependence of quality features. Shapiro-Wilk’s test was used to assess the normality of quantitative characteristics distribution. Since there were no normal distribution characteristics, the characteristics were analysed using non-parametric methods. Two groups were compared using U. Mann-Whitney’s test, and in case of three groups - ANOVA Kruskal-Wallis together’s test with post-hoc tests. Additionally, Spearman’s correlation coefficient was used. The study results of *p* <0.05 were regarded as statistically significant.

## Results

The study confirmed current demographic trends in regard to sex, as it included 213 (71%) women and 87 (29%) men. The median value of the overall result for AIS was 26 points, which is considered average in terms of illness acceptance. The median value of the impact of internal factors on pain control in case of BPCQ scale was 16 of 30 points, influence of physicians – 15 of 24 points, while random events – 14 of 24 points. The overall result for PRF scale proved that the respondents least expected to seek emotional support (5 of 12 possible points). No statistically significant differences were found between women and men in regard to the point values of the individual scales and subscales. The detailed data referring to the analysed subject for the whole group, and men and women are presented in Table 1.

**Table 1:** Mean values of AIS, BPCQ and PRF with regard to sex

	Women *N* = 213		Men *N* = 87		Total *N* = 300	
	$\overline{x}\pm S d$	Me	$\overline{x}\pm S d$	Me	$\overline{x}\pm S d$	Me
AIS	26 ± 8.97	26.0	26.7 ± 9.96	27.0	26.21 ± 9.26	26.0
BPCQ -internal factors	16.62 ± 4.5	16.0	16.28 ± 5.27	16.0	16.52 ± 4.73	16.0
BPCQ - physicians’ influence	15.08 ± 3.9	15.0	15.07 ± 4.29	15.0	15.07 ± 4.01	15.0
BPCQ - accidental incident	14.38 ± 4.33	14.0	13.57 ± 5.0	13.0	14.15 ± 4.54	14.0
PRF - expectation of disease explanation	7.98 ± 4.0	9.0	7.31 ± 4.06	8.0	7.79 ± 4.02	9.0
PRF - search for emotional support	5.34 ± 4.09	5.0	4.53 ± 3.75	4.0	5.11 ± 4.01	5.0
PRF - obtaining information about the subject of research and treatment	8.15 ± 3.86	10.0	7.9 ± 3.89	9.0	8.07 ± 3.87	9.0

Taking into consideration the mean values of the analysed scales in terms of the study group origin, the group of students of the University of Healthy Senior was characterised by the highest values, whereas, the residents of the nursing home - the lowest values. The figures are presented in Table 2. The statistical analysis proved statistically significant differences in case of AIS value between students of UHS and residents of NH (*p* < 0.001) and between students of UTA and residents of NH (*p* = 0.008). Analysing the influence of internal factors on pain control, statistically significant differences were noted between the same groups (*p* = 0.003 and *p* = 0.044, respectively). Significant differences regarding the influence of random events on pain control were also noted between students of UHS and residents of NH (*p* = 0.002).

**Table 2:** Mean values of AIS, BPCQ and PRF with regard to the study group

	UHS *N* = 100		UTA *N* = 100		PNH *N* = 100	
	$\overline{x}\pm S d$	Me	$\overline{x}\pm S d$	Me	$\overline{x}\pm S d$	Me
AIS	28.5 ± 9.36	30.0	26.91 ± 9.29	27.0	23.21 ± 8.37	23.0
BPCQ - internal factors	17.48 ± 4.84	17.0	16.86 ± 4.59	17.0	15.23 ± 4.51	15.0
BPCQ - physicians’influence	15.1 ± 4.24	15.0	14.89 ± 3.94	15.0	15.23 ± 3.89	15.0
BPCQ - accidental incidence	15.11 ± 4.16	15.0	14.49 ± 4.37	14.0	12.85 ± 4.81	13.0
PRF - expectation of disease explanation	7.71 ± 4.15	9.0	7.44 ± 4.16	8.0	8.21 ± 3.75	9.0
PRF- search for emotional support	4.53 ± 3.94	4.0	5.31 ± 3.88	6.0	5.48 ± 4.16	5.0
PRF - obtaining information about the subject of research and treatment	8.32 ± 4.06	10.0	7.8 ± 3.91	9.0	8.1 ± 3.63	9.0

The distribution of answers in the analysis of values of individual scales in terms of age of the respondents is presented in Table 3. The biggest group consisted of people aged 60–69 (over 50%). The youngest person was 60 years old, and the oldest – 98 years old. No statistically significant differences were observed between the age groups.

**Table 3:** Mean values of AIS, BPCQ and PRF in regard to respondents’ age

	60–69 years old *N* = 154		70–79 years old *N* = 102		80 and more years *N* = 44	
	$\overline{x}\pm S d$	Me	$\overline{x}\pm S d$	Me	$\overline{x}\pm S d$	Me
AIS	26.64 ± 9.26	27.0	26.32 ± 9.43	27.5	24.41 ± 8.8	24.0
BPCQ - internal factors	16.75 ± 4.96	16.0	16.12 ± 4.48	16.0	16.66 ± 4.49	17.0
BPCQ - physicians’influence	14.84 ± 3.9	15.0	15.16 ± 4.38	15.0	15.68 ± 3.5	15.0
BPCQ - accidental incidence	14.16 ± 4.43	14.0	14.48 ± 4.47	15.0	13.34 ± 5.06	13.0
PRF - expectation of disease explanation	7.99 ± 4.07	9.0	7.6 ± 4.08	8.5	7.5 ± 3.78	9.0
PRF - search for emotional support	5.18 ± 4.02	5.0	5.11 ± 4.11	4.0	4.86 ± 3.77	4.5
PRF - obtaining information about the subject of research and treatment	8.32 ± 3.83	10.0	8.03 ± 4.11	10.0	7.32 ± 3.34	7.0

Table 4 presents the AIS values in terms of the level of illness acceptance taking into account sex, age and the study group origin. There was no significant relation between the results of AIS and sex showed. However, it was found that belonging to the group (UHS, UTA, PNH) had a significant relationship with the results of AIS. Additionally, it was observed that the respondents from UHS (48%) showed the highest level of illness acceptance and the lowest level of illness acceptance was observed in the residents of NH (37%).

**Table 4:** A degree of illness acceptance among respondents with regard to sex, group affiliation, and age

		AIS result			*p*
		Low	Medium	High	
		*N* = 77	*N* = 119	*N* = 104	
Women	n	55	86	72	0.879
*N* = 213	%	25.82%	40.38%	33.80%	
Men	n	22	33	32	
*N* = 87	%	25.29%	37.93%	36.78%	
UHS	n	18	34	48	<0.001
*N* = 100	%	18.00%	34.00%	48.00%	
UTA	n	22	40	38	
*N* = 100	%	22.00%	40.00%	38.00%	
PNH	n	37	45	18	
*N* = 100	%	37.00%	45.00%	18.00%	
60–69	n	35	63	56	0.497
*N* = 154	%	22.73%	40.91%	36.36%	
70–79	n	27	38	37	
*N* = 102	%	26.47%	37.25%	36.27%	
80 and more	n	15	18	11	
*N* = 44	%	34.09%	40.91%	25.00%	

**Fig. 1:**
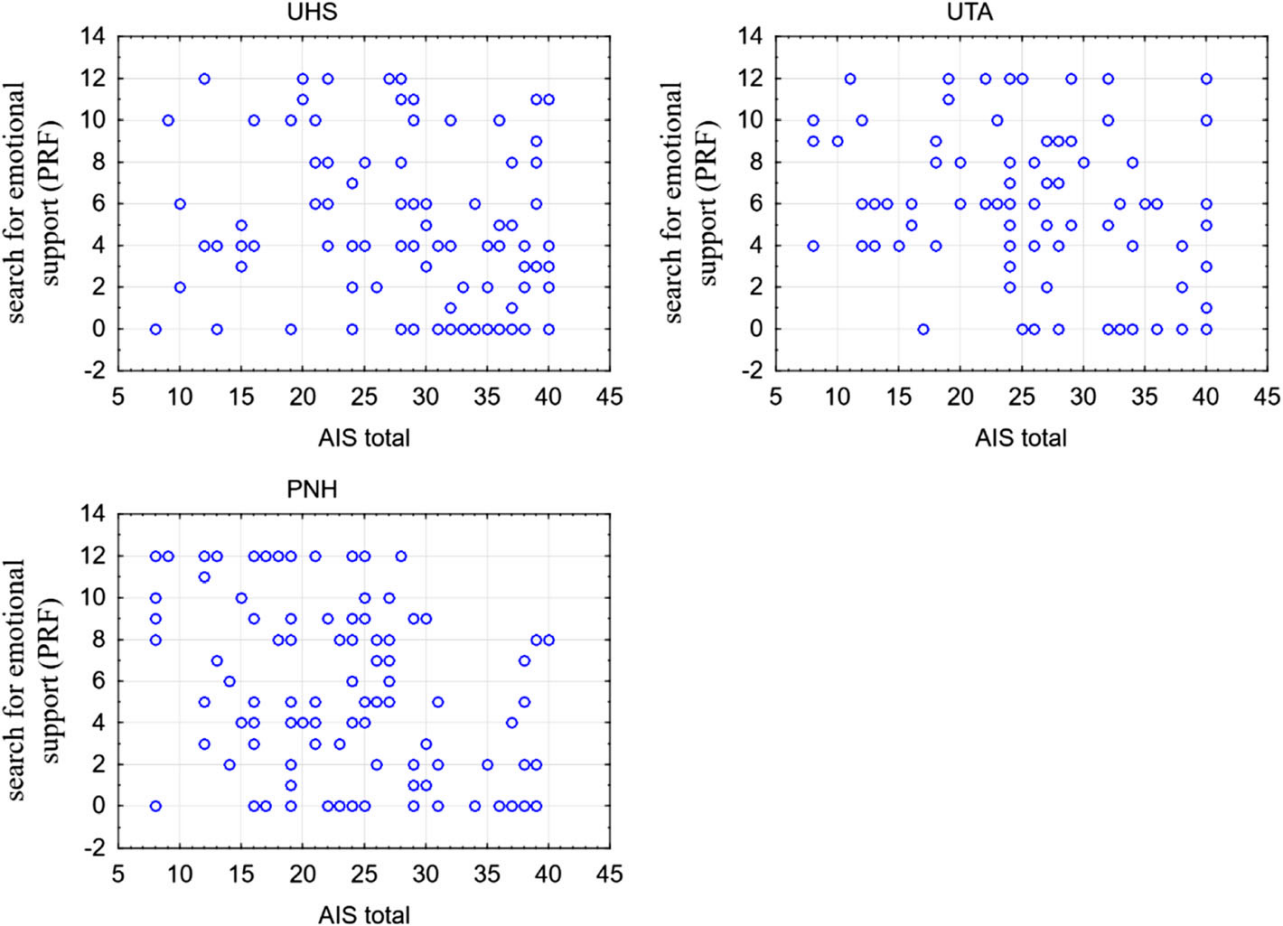
The scatterplots of AIS raw result and search for emotional support (PRF) according to the group affiliation

Apart from the overall results of the scales, it was decided to analyse the relationship between the raw results of individual scales, taking into account social and demographic features according to which the respondents were analysed. Taking into consideration the group origin, a negative correlation was noted between the AIS and the search for emotional support from a physician (PRF). The higher the value of AIS, the lower the point value in terms of seeking emotional support (PRF). In the group from UTA (*r* = −0.398) and NH (*r* = −0.337), this correlation was statistically significant (in both cases *p* < 0.001). In UHS group (*r* = −0.188), no statistically significant relationship was found, though the correlation was close to = 0.06. The analysed relationships are presented in Fig. 1.

In case of the analysis of the correlation between the raw result of AIS and seeking emotional support (PRF) taking into account age, the similar negative values were obtained. In the age groups 60–69 (*r* = −0.297; *p* < 0.001) and 70–79 (*r* = −0.382; *p* < 0.001), the correlations were statistically significant, while in the oldest age group, no significant relationship was found (*r* = −0.253; *p* = 0.097). The above correlation was also described in terms of sex.

**Fig. 2:**
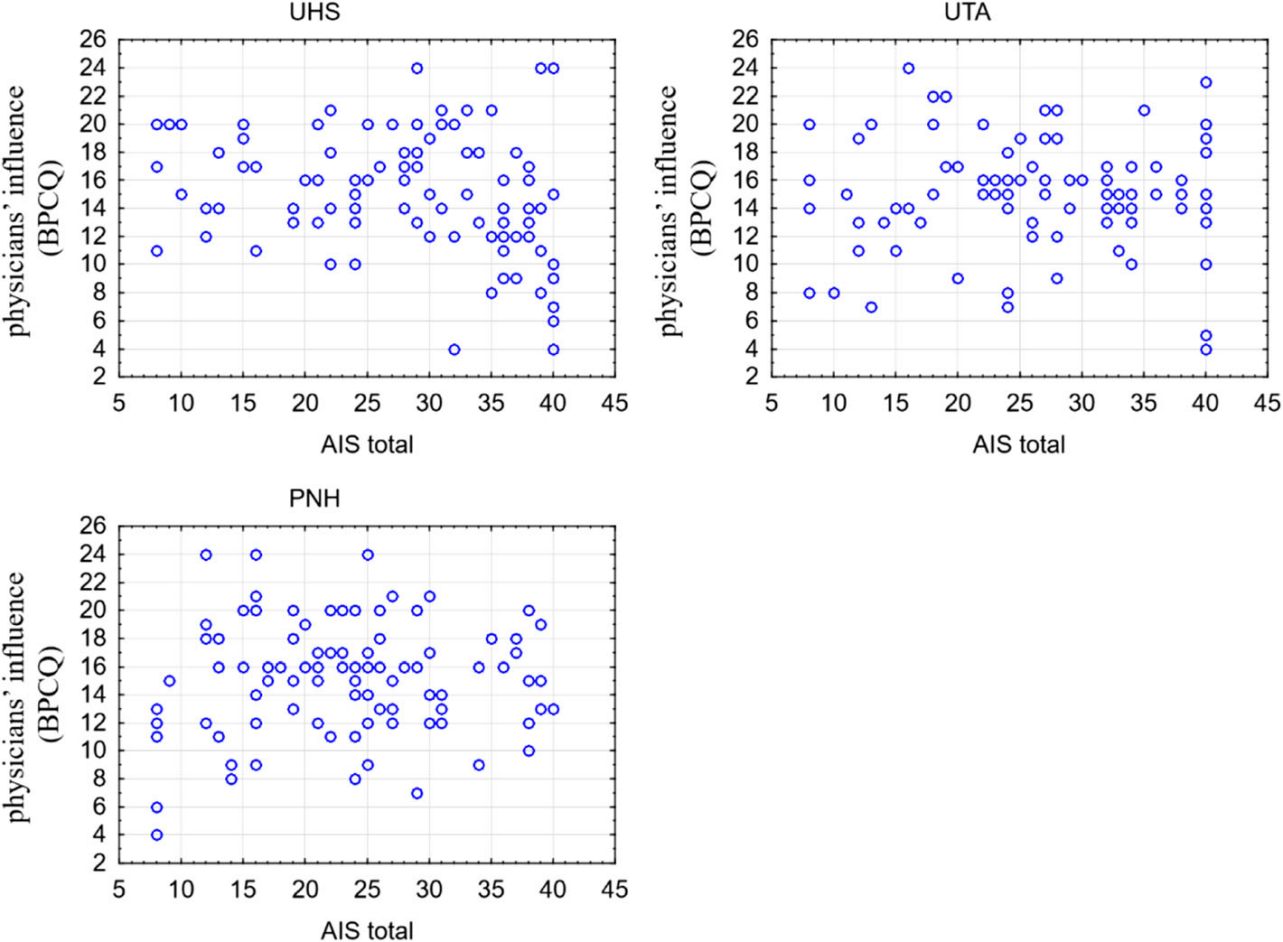
The scatterplots of AIS raw result and physicians’ influence on pain control according to the group affiliation

In the next stage, the correlation between the level of illness acceptance and influence of physicians was analysed (Fig. 2). A statistically significant relationship was found only in the group of UHS students (*r* = −0.240; *p* = 0.015). It was stated that the higher the level of illness acceptance among the respondents the lower their expectations in relation to the influence of physicians on their pain control. Such a relationship was not found in the other groups.

**Fig. 3:**
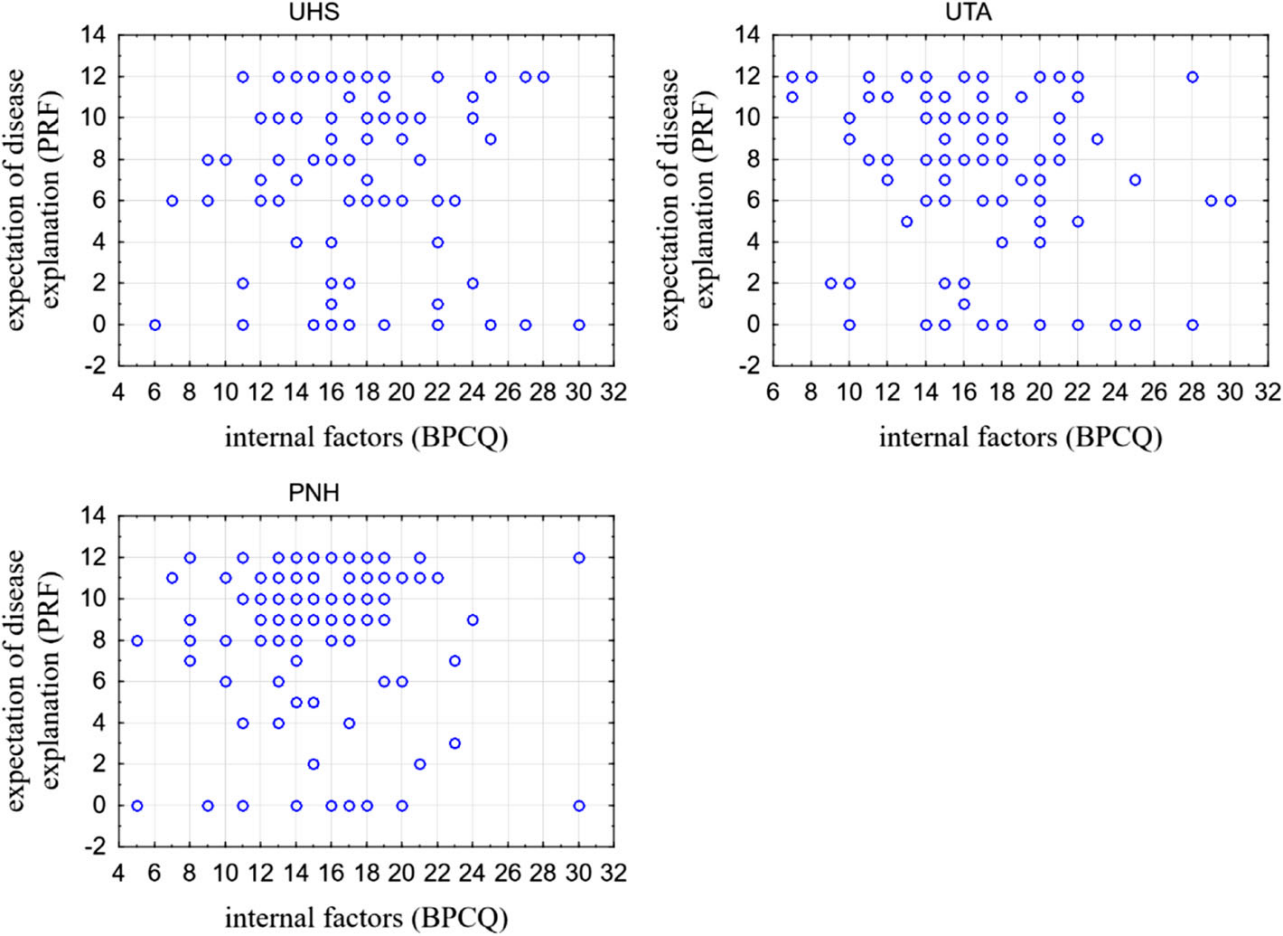
The scatterplots of the influence of internal factors on pain control (BPCQ) and expectation of a physician’s disease explanation (PRF) according to the group affiliation

One more relationship was analysed – between the influence of internal factors in case of pain control (BPCQ), and expectation for explaining the disease by the doctor. A significant relationship was found only in case of the respondents from UTA (*r* = −0.211; *p* = 0.034), which means that the higher the influence of internal factors on pain perception, the lower the expectations of UTA respondents for a physician in terms of explaining the disease process. No such relationship was found in the other groups. The detailed results are presented in Fig. 3.

## Discussion

### Illness acceptance

Adaptation to illness plays a key role in seniors’ adaptation to health limitations. Illness acceptance is one of the most important stages that apply to patients and their diseases; it maked the process of adaptation to the disease easier, i.e. the process in which a person adjusts to the new situation of living with an illness [18]. In our study, the average level of illness acceptance amounted to 28.5 ± 9.36 (Me = 30.0) in the study groups. A similar value, though a slightly lower, was determined by Kurpas et al. among chronically ill people aged over 60 (25.72 ± 8.22 (Me = 26)) [19]. In the study by Uchmanowicz et al. [20], the acceptance level of chronic obstructive pulmonary disease was 20.6 points in patients at the average age of 65.8 years, which meant a low level of illness acceptance. The identical value of AIS was also found in the study of Majda et al. [21]. Kupcewicz andAbramowicz noted slightly lower result (19 points) in patients with exacerbated chronic obstructive pulmonary disease (COPD) [22]. In patients with asthma, the indicator of illness acceptance was 29.4 points, while in patients dialysed due to renal failure – 25 points [23, 24]. The results of our study may suggest better quality of life and better mental adaptation to the disease of our respondents in comparison with the results of the results of the other studies presented above.

Illness acceptance is closely connected with and determines other psychological phenomena. In the study of Uchmanowicz et al. quoted before [20], illness acceptance was significantly correlated with the intensity of depressive symptoms. Kurpas et al. [19], Van Damme-Ostapowicz et al. [25] and Diener et al. [26] confirmed that a higher level of illness acceptance generated better quality of life, especially, in the physical and psychological aspect. Additionally, Van Damme-Ostapowicz et al. [25] showed in their studies that a higher level of illness acceptance determined a higher level of life satisfaction. Our study confirmed the correlation showing that a higher level of illness acceptance affects reduction of desire to search emotional support from physicians.

### Pain control

In our study, the level of pain control in relation to internal factors amounted to 16.52 points, depending on the influence of physicians – 15.07, and in regard to random events – 14.15. In the group of non-patients, Brown [27] reported a slightly lower result of BPCQ scale: 15.74 points in case of internal factors, 13.39 points in case of physicians’ influence and 10.77 points in terms of random events. In the study involving 90 people [28], Misterska et al. found very low average results, i.e., 3.26 points in relation to internal factors, 4.36 points in relation to physicians’ influence and 3.42 points in terms of random events. The results of studies by Wisniewska et al. [29] proved that patients attached the greatest significance in pain control to the influence of physicians and medical care. This value was 19.04 points and constituted 79.3% of the maximum value. Less significance lied in the influence of incidents – the average value was 16.85 points and constituted 70.2% of the maximum value. Studies of Zielazny et al. [30] proved that respondents had a differentiated approach to beliefs regarding the possible influence of factors on pain perception. The authors showed that the examined attributed the highest status to the influence of physicians on pain control. The results obtained in our study confirmed that in comparison with a few studies, where BPCQ scale was used as a study tool, our respondents believed that pain could be controlled using one factor.

### Patient’s list of expectations

Studies of Rotter et al. [31] proved that patients’ overall level of expectations regarding emotional support was low. Similar results were obtained in our study. The Lithuanian studies proved that expectation of emotional support was one of the four main factors affecting the Lithuanian patients’ expectations for primary health care [32]. In the studies by Kurpas et al., the percentage of people with a sense of emotional support received physicians of primary health care was similar to the percentage of those who believed they had no such support [33]. Additionally, Rotter’s et al. studies [31] proved that the elderly presented a higher level of expectations of emotional support from physicians of primary health care. There can be several reasons for this. Seniors, pensioners and annuitants usually have many health problems, they frequently suffer from pain and face difficult access to medical services, which triggers the search for emotional support. Lonely patients without emotional support from the relatives expect this from their family doctor, whom they often treat as a trustworthy person and a friend [31]. In the study, Glinska et al. [34] reported that patients aged 60–69 placed greater emphasis on care and compassion, a bit older patients expected mostly emotional support, whereas the oldest age group (80 years old and more) expected all types of help at the same level.

Completely different results were obtained in our study, especially, in the oldest age group. In the study group examined by Cieslak et al. [35], the expectations most frequently indicated by patients regarded being informed about the disease, its treatment and medical examinations. They expected emotional support the least. Unfortunately, because of not enough literature references about using the PRF questionnaire, more detailed analysis of the problem is not possible.

## Conclusions

1. Neither sex nor age played a significant role in acceptance of illness, control of pain and expectations for physicians.
2. Belonging to a group was a key variable affecting the relationship between the studied features.
3. The residents of the nursing home were the group negatively distinguishing in comparison to the other two study groups
4. The respondents compared to the other groups described in the literature, were characterised by relatively positive results of illness acceptance, pain control and expectations for physicians.

## Abbreviations

AIS: Acceptance of illness scale
BPCQ: the Beliefs about pain control questionnaire
COPD: Chronic obstructive pulmonary disease
PNH: Public nursing home
PRF: Patient request form
UHS: University of healthy senior
UTA: University of the third age
WHO: World health organization
